# The α-glucosidase inhibitor voglibose stimulates delayed gastric emptying in healthy subjects: a crossover study with a ^13^C breath test

**DOI:** 10.3164/jcbn.16-100

**Published:** 2017-04-20

**Authors:** Kenji Kanoshima, Mizue Matsuura, Megumi Kaai, Yumi Inoh, Kanji Ohkuma, Hiroshi Iida, Takashi Nonaka, Koji Fujita, Tomonori Ida, Akihiko Kusakabe, Atsushi Nakajima, Masahiko Inamori

**Affiliations:** 1 Department of Gastroenterology and Hepatology, Yokohama City University School of Medicine, 3-9 Fukuura, Kanazawa-ku, Yokohama 236-0004, Japan; 2 Office of Postgraduate Medical Education, Yokohama City University School of Medicine, 3-9 Fukuura, Kanazawa-ku, Yokohama 236-0004, Japan; 3 Department of Medical Education, Yokohama City University School of Medicine, 3-9 Fukuura, Kanazawa-ku, Yokohama 236-0004, Japan

**Keywords:** gastric emptying, breath test, voglibose

## Abstract

The gastrointestinal effects of α-glucosidase inhibitors have not been sufficiently investigated. The aim of this study was to determine whether a single dose of pre-prandial voglibose might affect the rate of gastric emptying, determined using the ^13^C breath test. Ten healthy male volunteers participated in this randomized, two-way crossover study. The subjects fasted overnight and received 0.2 mg voglibose or a placebo 2 h before a test meal. They were then served a liquid test meal consisting of 200 kcal per 200 ml that contained 100 mg ^13^C-acetate. Breath samples were collected under both conditions until 150 min after the meal. A comparison of the control and voglibose conditions revealed that for gastric emptying rates (with values expressed as median: range), T_1/2_ [(87.9: 78.0–104.9 min) vs (88.4: 74.3–106.3 min), *p* = 1], T_lag_ [(47.1: 39.6–60.1 min) vs (45.4: 31.2–63.3 min), *p* = 0.432], β [(1.89: 1.68–2.18) vs (1.90: 1.35–2.15), *p* = 0.846] and κ [(0.81: 0.71–0.98) vs (0.81: 0.50–0.94), *p* = 0.922] did not significantly differ between conditions. A significant difference between the control and voglibose conditions was found for the GEC [(4.28: 4.09–4.44) vs (4.06: 3.69–4.50), *p* = 0.0138]. In conclusion, this study demonstrated that the ingestion of oral voglibose led to delayed gastric emptying of a liquid meal.

## Introduction

Many drugs are available for diabetic patients, but their effects on gastric emptying are poorly understood. Voglibose is an inhibitor of α-glucosidase, which is an enzyme secreted from the brush border of the small intestine that induces maldigestion of disaccharides and absorption of glucagon-like peptide 1 (GLP-1)-rich intestinal segments.^(1)^

GLP-1 is an incretin hormone derived from post-translational processing of pre-proglucagon that is secreted from the intestine into the circulation in response to food ingestion.^(2–5)^ Together with gastric inhibitory polypeptide (GIP), GLP-1 helps manage glycemic control by regulating insulin and glucagon release, slowing gastric emptying and reducing caloric intake.^(6–8)^

When voglibose and the α-glucosidase inhibitor acarbose are combined with sucrose, they elevate and extend GLP-1 release in healthy volunteers in the same manner as type 2 diabetic patients.^(9–11)^ According to a previous study, eight healthy subjects exhibited a deceleration of gastric emptying measured by paracetamol absorption after the administration of acarbose.^(12)^ Delays of gastric emptying could result from an increase in GLP-1 secretion due to its ability to slow gastric emptying.^(13–15)^

In the present study, the pharmacological effects of a single dose of pre-prandial voglibose on the rate of liquid gastric emptying were examined in healthy volunteers using a ^13^C-acetic acid breath test.

## Materials and Methods

### Ethics

The study was conducted in accordance with the principles of the Declaration of Helsinki. The study protocol using the breath test was approved by the Ethics Committee of Yokohama City University School of Medicine (No. A110929010).

### Subjects

The ten subjects included asymptomatic male volunteers (median age 24 years, range 21–42 years). The height and weight of the subjects were as follows: median height, 168.3 cm; height range, 160–182 cm; median weight, 63.7 kg; and weight range, 42–98 kg. None of the subjects were habitual drinkers. All subjects were non-smokers, and none had a history of gastrointestinal disease or abdominal surgery. None of the subjects were taking any routine medication at the time of the study.

### Methods

Ten subjects participated in this randomized, two-way crossover study. After overnight fasting (at least 8 h), they received 0.2 mg voglibose orally (voglibose condition) or a placebo 2 h before the test meal (placebo condition) in a random sequence. The 2 test conditions were separated by a washout period of at least 7 days. The test meal consisted of a 200 kcal per 200 ml liquid meal (Racol with milk flavor, Otsuka Pharmaceutical, Co., Ltd., Tokyo, Japan) containing 100 mg of ^13^C-acetic acid (Cambridge Isotope Laboratories, Inc., Andover, MA), and the subjects were requested to consume the meal within 5 min.

Gastric emptying was measured using the ^13^C-acetic acid breath test while the subjects were seated. Breath samples were collected in air bags at baseline (before the test meal) and at 5, 10, 15, 20, 30, 40, 50, 60, 75, 90, 105, 120, 135 and 150 min after the test meal was ingested. The ^13^CO_2_/^12^CO_2_ ratio in collected breath samples was determined as the difference from baseline using non-dispersive infrared spectrophotometry (POCone, Otsuka Electronics Co., Ltd., Osaka, Japan).

### Data analysis

In accordance with the method reported by Ghoos *et al.*,^(16)^ the percentage of ^13^CO_2_ recovery in expired breaths per hour (percent dose per hour) against time was fit to the formula y(t) = at^b^e^−ct^ using non-linear regression analysis, where y is the percentage of ^13^C excretion in the breath per hour, t represents time in hours, and a, b, and c are constants. The time course of cumulative ^13^CO_2_ recovery in expired breaths was fit to another formula, z(t) = m(1 – e^−kt^)β, where z is the percentage of the cumulative ^13^C excretion in expired breaths and also an integral of y(t), m is the cumulative ^13^CO_2_ recovery at an infinite time, and β and κ are regression-estimated constants. β and κ were determined using the mathematical curve-fitting technique. A larger β indicates slower emptying in the early phase, and a larger κ indicates faster emptying in the later phase, whereas smaller values correspond to faster and slower emptying during those phases, respectively. The time required for 50% emptying of the labeled meal (T_1/2_), the analog to the scintigraphy lag time for 10% emptying of the labeled meal (T_lag_) and the gastric emptying coefficient (GEC) were calculated as overall measures of gastric emptying: T_1/2_ = −[ln(1 − 2^−1/^β)]/κ, T_lag_ = (lnβ)/κ and GEC = ln(a). These parameters were calculated using the Solver procedure in Excel 2010 (Microsoft Corp., Redmond, WA).

### Statistical methods

Statistical evaluation was performed using the Wilcoxon’s signed-rank test. The level of significance was set at *p*<0.05. We previously estimated that 90% of the subjects would show delayed liquid gastric emptying in the voglibose condition compared to the placebo condition. All statistical analyses were performed with EZR (Saitama Medical Center, Jichi Medical University, Saitama, Japan), which is a graphical user interface for R (The R Foundation for Statistical Computing, Vienna, Austria). More precisely, EZR is a modified version of R commander designed to add statistical functions that are frequently used in biostatistics.^(17)^

## Results

All 10 subjects completed this study, and no adverse events were reported. Table 1 presents the study results. No significant differences were observed in T_1/2_ [(87.9: 78.0–104.9 min) vs (88.4: 74.3–106.3 min), *p* = 1], T_lag_ [(47.1: 39.6–60.1 min) vs (45.4: 31.2–63.3 min), *p* = 0.432], β [(1.89: 1.68–2.18) vs (1.90: 1.35–2.15), *p* = 0.846] and κ [(0.81: 0.71–0.98) vs (0.81: 0.50–0.94), *p* = 0.922] (median: range, placebo vs voglibose) between the placebo and experimental conditions. A comparison of GECs for the control and voglibose groups revealed significant delay in the voglibose group [(4.28: 4.09–4.44) vs (4.06: 3.69–4.50), *p* = 0.0138].

**Fig. 1:**
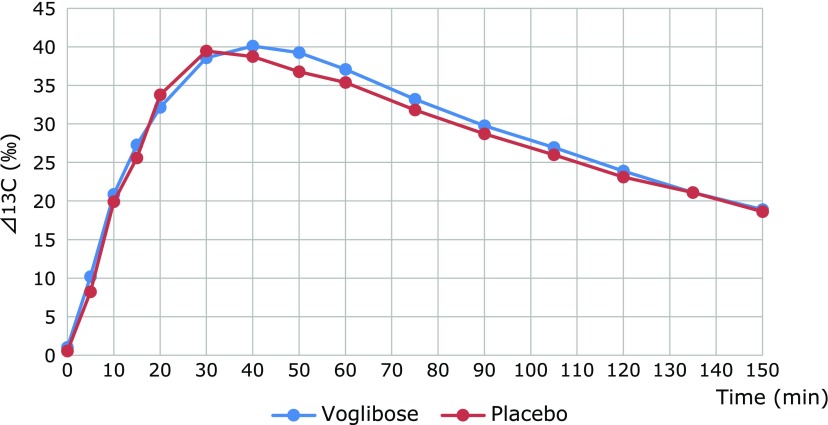
Time course of Δ^13^CO_2_ (‰). We demonstrate the data of mean time course changes of ^13^CO_2_ in voglibose (blue) and placebo (red) groups as Fig. 1. This figure revealed delayed gastric emptying visually in the voglibose group.

We demonstrate the data of mean time course changes of ^13^CO_2_ in both groups as Fig. 1. Fig. 1 revealed delayed gastric emptying visually in the voglibose group.

These results indicated that voglibose had an effect on the rate of liquid gastric emptying.

## Discussion

In this study, we examined changes in the rate of liquid gastric emptying after a single dose of pre-prandial voglibose (0.2 mg) during the first 2.5 h after ingestion of a liquid meal in healthy volunteers. Significant differences were observed in the GEC of the liquid gastric emptying parameters measured with a ^13^C-acetic acid breath test between the two test conditions (i.e., voglibose before the meal and the test meal alone). These results indicate that voglibose delayed the rate of liquid gastric emptying.

Unfortunately, few reports have been published regarding the effects of voglibose on gastric emptying. Ranganath *et al.*^(18)^ suggested that another α-glucosidase inhibitor, acarbose, was associated with the reduction in gastric emptying in healthy subjects who received an oral sucrose load. However, Hücking *et al.*^(19)^ reported that ingestion of acarbose with a mixed test meal failed to enhance GLP-1 release and did not influence gastric emptying in hyperglycemic patients with type 2 diabetes. Differences in conditions were present, including the selection of subjects (type 2 diabetic patients rather than healthy young volunteers), the choice of the meal (a mixed, breakfast-type meal vs a liquid 100 g sucrose load) and the method of determining gastric emptying (paracetamol absorption vs ^13^C-octanoic acid breath test),^(16,20,21)^ and other factors likely contributed to the discrepancy in the results.^(19)^

The present study had some limitations. First, the number of subjects included was small. Second, this study was performed in healthy subjects who had normal gastric contractile function. In addition, we did not assess the actual serum GLP-1 concentrations enhanced by voglibose in these subjects.

This study was conducted in healthy, normoglycemic male subjects, which limited the extent to which the data can be extrapolated to patients with type 2 diabetes. As mentioned above, the pharmacokinetic and pharmacodynamic profiles of voglibose have been reported to be similar in healthy individuals and in those with type 2 diabetes.^(22,23)^ However, gastric emptying rates in the type 2 diabetes population have been reported to be delayed, unchanged, or accelerated.^(24–28)^ Determination of gastric emptying rates in healthy individuals might be advantageous for acquiring knowledge of the natural characteristics of pharmaceutical preparations in contrast to those of type 2 diabetics, who might exhibit increased heterogeneity in their rates of gastric emptying.

Treatment of type 2 diabetic patients is not only medication but also exercise therapy. Recently, interesting knowledge about the effect of exercise therapy on gastric emptying has been reported.^(29)^ In clinical settings, we should evaluate comprehensively about gastric emptying of diabetic patients.

Finally, the results of this study might be beneficial for functional dyspepsia patients who often exhibit rapid gastric emptying. A recent study reported that rapid gastric emptying might be a more important factor than delayed gastric emptying in patients with functional dyspepsia.^(30)^

In conclusion, voglibose significantly affected processing of a liquid meal in healthy individuals. Therefore, a deceleration of gastric emptying was observed in healthy volunteers that contributed to the mechanism of α-glucosidase inhibitors in this group.

## Figures and Tables

**Table 1:** A comparison of breath test parameters for the placebo and voglibose groups

Parameter	Placebo group	Voglibose group	*p* value
T_1/2_	1.46 (1.30–1.75)	1.47 (1.24–1.83)	1
T_lag_	0.78 (0.66–1.00)	0.76 (0.60–1.05)	0.432
β	1.89 (1.68–2.18)	1.90 (1.35–2.83)	0.86
κ	0.81 (0.71–0.97)	0.81 (0.50–1.06)	0.922
GEC	4.28 (4.09–4.44)	4.06 (3.69–4.50)	0.0138

All parameter values are expressed as median (range). Abbreviations: T_1/2_, the time required to empty 50% of the labeled meal (min); Tlag, the analog to scintigraphy lag time for emptying 10% of the labeled meal; β and κ, the regression-estimated constants; GEC, the gastric emptying coefficient.
